# Deciphering the nonsense-mediated mRNA decay pathway to identify cancer cell vulnerabilities for effective cancer therapy

**DOI:** 10.1186/s13046-021-02192-2

**Published:** 2021-12-01

**Authors:** Roberta Bongiorno, Mario Paolo Colombo, Daniele Lecis

**Affiliations:** Department of Research, Fondazione IRCCS Istituto Nazionale dei Tumori, Via Amadeo 42, 20133 Milan, Italy

**Keywords:** Neoantigens, Immune checkpoint inhibitors, Nonsense mutations, Combination therapy

## Abstract

Nonsense-mediated mRNA decay (NMD) is a highly conserved cellular surveillance mechanism, commonly studied for its role in mRNA quality control because of its capacity of degrading mutated mRNAs that would produce truncated proteins. However, recent studies have proven that NMD hides more complex tasks involved in a plethora of cellular activities. Indeed, it can control the stability of mutated as well as non-mutated transcripts, tuning transcriptome regulation. NMD not only displays a pivotal role in cell physiology but also in a number of genetic diseases. In cancer, the activity of this pathway is extremely complex and it is endowed with both pro-tumor and tumor suppressor functions, likely depending on the genetic context and tumor microenvironment. NMD inhibition has been tested in pre-clinical studies showing favored production of neoantigens by cancer cells, which can stimulate the triggering of an anti-tumor immune response. At the same time, NMD inhibition could result in a pro-tumor effect, increasing cancer cell adaptation to stress. Since several NMD inhibitors are already available in the clinic to treat genetic diseases, these compounds could be redirected to treat cancer patients, pending the comprehension of these variegated NMD regulation mechanisms. Ideally, an effective strategy should exploit the anti-tumor advantages of NMD inhibition and simultaneously preserve its intrinsic tumor suppressor functions. The targeting of NMD could provide a new therapeutic opportunity, increasing the immunogenicity of tumors and potentially boosting the efficacy of the immunotherapy agents now available for cancer treatment.

## Background

Nonsense-mediated mRNA decay (NMD) pathway is a protection mechanism of eukaryotic cells, being responsible for the identification and degradation of mRNAs with aberrant mutations. In this way, NMD prevents the production of altered polypeptides with potentially toxic dominant-negative activity [1]. Although this is the standard definition of NMD, its role is far from being just that. Indeed, the NMD pathway is also involved in the fine physiological regulation of the transcriptome thus playing a crucial role in stress adaptation and differentiation. NMD is known to contribute to the onset of genetic pathologies in the presence of nonsense mutations [2]. Moreover, several new findings support the notion that the NMD pathway plays an important role also in defining cancer cell outcome, being both favorable and unfavorable depending on tumor type and microenvironment context [3, 4]. In this review, we focus on this double effect that the NMD endows in cancer and the potential therapeutic application of NMD inhibitors.

## Main text

### NMD activation mechanisms

The detection of a transcript bearing a premature termination codon (PTC) is the best characterized NMD function. However, the NMD machinery is not only able to target mutated disease-causing mRNAs, but also to physiologically regulate about 10% of the transcriptome of human cells [5]. The NMD molecular pathway involves the recruitment of the kinase SMG1 that in turn is responsible for the activation of the key NMD protein, UPF1 [6]. In detail, SMG1 and UPF1 interact with the translation termination factor eRF1 and eRF3, and form the SMG1-Upf1-eRF1-eRF3 (SURF) complex which then promotes the activating phosphorylation of UPF1 by SMG1 [7]. Then, phosphorylated UPF1 acts as a platform for the recruitment of the endonucleases responsible for mRNA degradation [8]. The way by which NMD discriminates its targets is still not entirely clear, but, currently, it is thought that cells employ two main mechanisms to select the mRNA target, one being exon junction complex (EJC)-dependent and the other EJC-independent.

### EJC-dependent and -independent NMD

The EJC-dependent NMD has been considered the only NMD activation mechanism for a long time. According to this mechanism, the detection of the mRNA target occurs when a PTC is coupled with the splicing machinery [9]. In fact, when a transcript undergoes splicing, a large protein complex, the EJC, is recruited 20-24 nucleotides upstream the splicing site [10]. During translation, ribosomes normally remove EJC in order to completely translate the transcript, but an EJC present after a stop codon will not be removed and will become the triggering signal for NMD activation as demonstrated in a *Xenopus* model [10]. In agreement with this idea, PTCs localized in the last exon are not recognized by NMD. In the EJC-dependent NMD, two other members of the Upf family, Upf2 and Upf3, are also recruited by the SURF complex making PTC recognition more efficient [11]. In particular, Upf3 was shown to directly interact with eRF3 in yeast [12].

Nevertheless, several pieces of evidence show that also transcripts not containing an EJC can be targeted by NMD. Indeed, mRNAs without introns can similarly be recognized by NMD and the artificial localization of a proximal intron downstream a physiological stop codon does not affect the stability of the transcript, and the splicing process correctly occurs [13, 14]. Taking into account these observations, the EJC-independent model was proposed. According to this, PTC discrimination depends on a competition mechanism in which eRF3 interacts alternatively with Upf1 or with the cytoplasmic polyadenylate-binding protein (PABC1), depending on PTC position [15]. Generally, a physiological stop codon is localized near the poly(A) tail, favoring eRF3-PABP interaction and so determining the normal termination of translation [16]. On the contrary, a PTC being physically distant from the 3′-UTR region makes PABP-eRF3 interaction difficult and favors the recruitment of Upf1 with consequent mRNA decay, at least in yeasts [17, 18]. Although this mechanism was also confirmed by employing human cell lines [19], more recent evidence obtained through genomics analyses suggests that a long 3’UTR could trigger NMD only in particular cases and be not so relevant in human NMD [20]. Moreover, several mammalian cell mechanisms are able to protect long 3’UTR from NMD. This is the case of PTBP1 [21] and hnRNP L [22] which both interact with specific regions near the termination codon and inhibit the recruitment of UPF1, hence preventing mRNA degradation.

A prominent role of NMD pathway, in addition to the recognition of nonsense transcripts, is to act as a post-transcriptional regulator of entire groups of genes further indicating that NMD is able to target also not mutated mRNAs as shown in yeasts [23]. In this case, the EJC-independent mechanism was proposed to explain how NMD is able to physiologically regulate transcriptome. A long 3’UTR (> 1 kb) could reduce the efficiency of eRF3-PABP interaction and favor Upf1 binding, thus increasing the chance of Upf1 activating phosphorylation [2]. However, recent findings strongly support the notion that the activation of NMD is not caused by 3’UTR length per se, but by the increased presence of exon junctions in longer 3’UTR [20, 24]. Many works investigated the mechanisms by which NMD regulates the stability also of non-mutated mRNAs and described several “rules” that could expand the concept of EJC-independent mechanism. These works detailed how the regulation of a physiological transcript decay depends on the balance between NMD inhibitory cis-agents elements and NMD activating features intrinsic of the transcript. Accordingly, an upstream open reading frame (uORF) increases sensitivity to NMD of mRNAs [25] not displaying an exon-exon junction in the 3’UTR [24]. Retained introns and the presence of exons in 3’UTR are all mechanisms associated to increased NMD activity [24]. Moreover, mRNAs generated by alternative splicing (AS) are characterized by different half-lives due to NMD-dependent degradation. This AS coupled with NMD (AS-NMD) was first described in mouse cells [26] and then confirmed in *C. elegans* [27], finding that this process is regulated by exons containing “ultraconserved elements” and it is highly preserved among different species [28].

### Role of NMD in physiological conditions and in stress adaptation

The NMD pathway is able to guarantee a fine quality control of transcriptome with a precise timing of expression of NMD targets. This allows cells to adapt in response to stress conditions or modify transcriptome during development. The importance of NMD is evidenced by the fact that the knockout of NMD components, such as Upf1 and Smg1, causes lethality in mice and has been associated with intellectual disability in humans [29]. Indeed, the NMD-mediated regulation of gene expression is important for the differentiation of several cell types, such as neurons and embryonic stem cells (ESCs), where NMD acts as regulator of key grow factors (e.g. TGFβ and BMP) [30]. NMD results fundamental also during lymphocyte development, during which T-cell receptor (TCR) programmed rearrangement generates various unproductive gene products bearing a PTC. Aberrations in the NMD process cause an increase of nonsense TCR transcripts that result toxic for cells [31].

The NMD pathway regulates a set of genes involved in the integrated stress response (ISR) and in the unfolded protein response (UPR), preventing their activation in the presence of low levels of stimuli [32]. On the other hand, both ISR and UPR, activated in the presence of medium-high stresses, induce eukaryotic translation initiation factor 2α (eIF2α) phosphorylation which decreases general protein synthesis and suppresses NMD [32]. The mechanisms underlying eIF2α phosphorylation-dependent NMD inhibition are still largely unknown and cannot be simply explained by the overall reduction of protein translation which indeed reduces the expression levels also of NMD factors. Accordingly, it has been proposed that phosphorylation of eIF2α could also inhibit NMD through the relocalization of NMD mediators in stress granules, resulting so physically distant from their mRNA targets. Hence, in the presence of high levels of cellular stress, NMD is inhibited and this allows the stabilization of stress mediator transcripts, although, in theory, the phosphorylation of eIF2α should prevent their translation. Nonetheless, it was shown that this is not the case because, despite the global reduction of protein synthesis, several stress mediators such as ATF-4, ATF-3 and CHOP are indeed translated upon cellular stress [33], and display increased transcript stability as a consequence of NMD inhibition and through specific escape mechanisms involving for example a different uORF [34]. This event leads to increased levels of stress response mediators, such as cysteine/glutamate exchanger SLC7A11, which is involved in the production of glutathione to reduce reactive oxygen species (ROS) level, and IRE1α, a master regulator of UPR, whose transcript is normally recognized by NMD likely due to the presence of a long 3’UTR [35]. Moreover, in the case of prolonged or excessively high stress, to which adaptation is precluded, Upf1 is cleaved by caspases, generating a peptide with a dominant negative function on NMD activation. This event causes the upregulation of proapoptotic NMD targets such as BAK1, DAP3, DUSTP2 and GADD45α [36, 37]. Cellular stresses that activate ISR and inhibit NMD pathway include hypoxia and production of ROS that are also distinctive elements of tumor environment, so determining a possible inhibition of NMD in tumor cells.

### Pro-tumor functions of NMD

NMD detects PTCs, which can be caused by frameshift indel mutations, and results in the degradation of mutated mRNAs potentially encoding novel immunogenic peptides. This concept implies that the inhibition of the NMD machinery could increase the production of neoantigens especially in unstable and error-prone cells, such as tumor cells. Accordingly, it has been shown that the genetic inhibition of Upf2 or Smg1 resulted in immune-dependent reduction of tumor growth in syngenic mouse models [38]. On the same line, in vitro silencing of UPF1 caused the production of a large number of neoantigens potentially able to activate T-cell responses [39]. Neoantigen expression in osteosarcoma was shown to be reduced by NMD ultimately resulting in an accumulation of genomic alterations not paralleled by immune infiltration [40]. In the immunotherapy era, these observations prompt the opportunity to combine NMD inhibitors, already available for the treatment of several genetic diseases, with immune checkpoint inhibitors exploiting the potential increase of the antigenic repertoire of target tumors. Although not all mutations are immunogenic, frameshift mutations are usually associated with the highest immunogenicity. Further supporting the pivotal role of NMD in preventing the expression of mutated mRNA, it was shown that expressed indel mutations eventually translated into detectable proteins were indeed enriched in genomic regions likely to escape NMD [40]. Hence, analysis of frameshift mutations potentially not detected by NMD could be employed to stratify patients more likely to respond to immune checkpoint-based therapy [41], an approach which was shown to be predictive of therapy response [40, 41]. Due to the capacity of NMD to reduce the expression levels of mutated genes, it is not surprising that NMD components are expressed at higher levels in microsatellite instability (MSI) tumors [42] which are characterized by defects in the DNA mismatch repair (MMR) system [3] and have been the focus of intense investigation [42–44]. In these settings, the pharmacological inhibition of NMD could favor HLA class I-mediated presentation of indel neoepitopes which could be exploited to trigger a CD8 T-cell response [45]. Accordingly, NMD activity is a negative predictive factor of immune response against MSI colorectal cancer (CRC; [42]).

Along with the effect on the expression of immunogenic peptides, the inhibition of NMD allows the expression of different toxic mutated proteins and results in an anti-tumor effect. The inhibition of NMD could hence represent a strategy to stimulate the re-expression of a plethora of NMD-suppressed genes [44, 46] to inhibit tumor growth. This approach has been exploited in preclinical settings also to identify mutations targeted by NMD and to characterize novel tumor-suppressor genes [46]. By following the latter approach, a plethora of genes both mutated and not mutated [47] have been shown to be degraded in an NMD-dependent manner. Nevertheless, NMD-mediated degradation of mutated mRNA is strictly selective [43, 48].

### NMD as a tumor suppressor interconnected with the tumor microenvironment (TME)

As mentioned above, NMD can favor cancer cells by preventing the expression of mutated antigens with immunogenic activity, but it is also endowed with opposite activities with tumor-suppressor functions. For example, mutated BRCA1, which is responsible for breast and ovarian familiar cancer, can display dominant negative activities when prematurely truncated [49]. Nonetheless, by comparing the expression levels of wild type and mutated alleles within the same patient, it was shown that mutated transcripts levels are lower compared to the wild-type counterpart. The NMD was shown to be responsible for the selective degradation of the mutated allele transcript [50] hence acting as a surveillance mechanism.

**Fig. 1:**
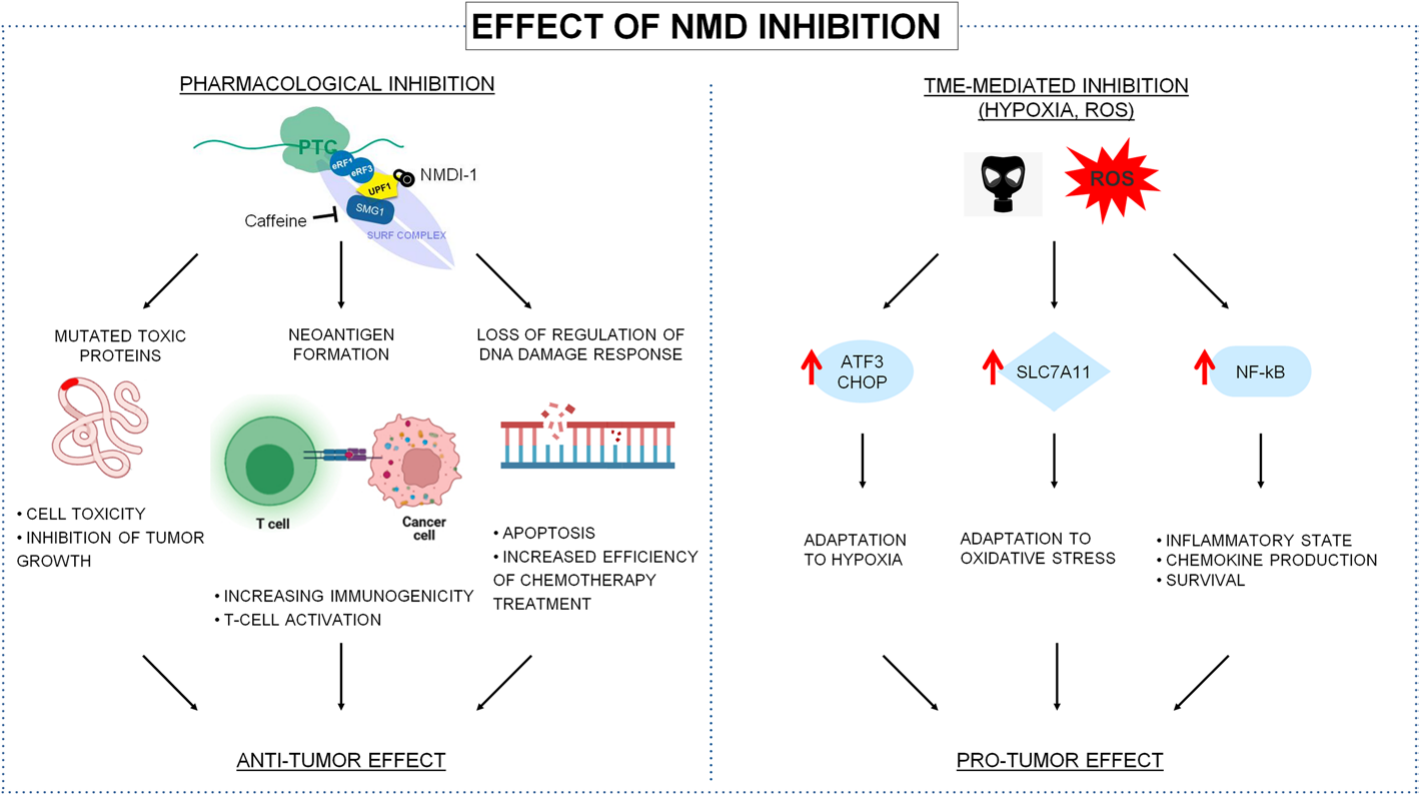
Pro- and anti-tumor effects of NMD inhibition. NMD has been shown to play a critical role in tumors, displaying, at the same time, a pro-tumor and tumor suppressor activity, depending on the genetic context and tumor microenvironment. NMD, degrading mRNAs with a PTC, protects cells from the formation of potentially toxic proteins. Indeed NMD pathway inhibition results in tumor cell toxicity due to the accumulation of mutated protein. Moreover, immunogenic peptides, derived from these proteins, could act as neoantigens able to activate T-cell response against tumor cells, inducing so an immune-dependent reduction of tumor growth. Furthermore, NMD physiologically regulates genes involved in DNA damage response, so NMD inhibition results in a reduction of tumor capacity to respond to DNA damage and in a greater sensitivity to chemotherapy. On the other hand, for its tumor-suppressor role, NMD is often inhibited by microenvironmental cues, as hypoxia or oxidative stress, determining an increased expression of ISR components, like ATF3 and CHOP, or antioxidant agent such as SLC7A11. In this way, NMD inhibition protects cancer cells from elevated level of ROS and reduced oxygen concentration allowing tumor adaptation to stress conditions. Moreover, impaired NMD was found to favor activation of the NF-kB pathway so inducing an inflammatory state and favoring tumor cell survival. Adapted from BioRender.com

In agreement with an anti-tumor role of NMD, there are several findings showing that this pathway is repressed along with tumor evolution and development. The inhibition is especially triggered by microenvironmental cues as hypoxia [32] which represses the expression of NMD components in an eIF2α-dependent manner, ultimately favoring the expression of ATF-4, ATF-3, and CHOP (Fig. 1). These mediators are components of the ISR and favor tumor adaptation to low oxygen levels, also favoring the induction of autophagy [51] which represents a cancer cell resistant state [52]. Microenvironment-dependent inhibition of NMD has been shown to promote also the expression of SLC7A11 [53] which can protect cancer cells from oxidative stress. SLC7A11/xCT allows cystine intake by cells and it is hence essential for glutathione synthesis which in turn constitutes an antioxidant defense. Not surprisingly, SLC7A11 is often found over-expressed in cancer cells and has been proposed as a novel target in cancer therapy [54].

NMD is hence inhibited by TME-induced stress to favor tumor adaptation to the surrounding hostile conditions. However, by causing the degradation of a plethora of mutated mRNA and potentially controlling about one third of all alternatively spliced mRNAs [55], NMD in turn has the potential to profoundly mold the TME. The last aspect has still been poorly investigated in cancer, but could further complicate the already intricated connection between cancer cells and TME. For example, a recent work shows mutation in UPF1 in 13 out of 15 inflammatory myofibroblastic tumor (IMT) samples [56]. Impaired NMD was found to promote the accumulation of NIK that is a potent activator of the non-canonical NF-kB pathway and stimulator of several pro-inflammatory cytokines such as TNF [57]. NIK is usually degraded in a continuous fashion by cIAP1 and cIAP2 [58] in order to block the activation of its downstream pathway, but mutations or silencing of UPF1 were shown to aberrantly cause the accumulation of NIK resulting in chemokine production and massive immune infiltration in lung IMT [56]. NMD regulates NF-kB also at different levels and SMG7 was identified as a determinant of resistance to TNF-induced extrinsic apoptosis induction [59]. Accordingly, its depletion was shown to prevent sensitivity to TNF and favor activation of the NF-kB pathway via reduction of tumor suppressor cylindromatosis CYLD [60], a deubiquitinase that cleaves polyubiquitin chains and is involved in TNF-induced necroptosis.

### Impact of NMD on cancer treatment efficacy

Since NMD regulates the transcription levels of many pathways in normal and cancer cells, it is easy to deduce that NMD could also impact a plethora of cell responses and ultimately also influence anti-cancer therapy efficacy. Traditional chemotherapy relies on the activation of apoptosis which is negatively regulated by NMD [36]. Interestingly, apoptosis in turn is able to inhibit NMD through caspase-9-dependent cleavage of UPF1. These findings support the idea that NMD and apoptosis balance is finely regulated. Inhibition of NMD was shown to promote the in vitro efficacy of doxorubicin in human cancer cell lines [36] and NMD was proved to regulate a number of genes involved in DNA damage response. Since traditional chemotherapy is effective by inducing DNA damage, this provides an additional mechanism by which NMD can influence cancer outcome upon chemotherapy administration. For example, p53 activation, which occurs in response to genotoxic stress induced by chemotherapy, is essential for cell cycle arrest and allows DNA damage repair. It has been shown that p53 expression depends on the NMD mediator SMG1 [61], which hence influences chemotherapy efficacy via p53. Moreover, p53 is often mutated in cancer cells and it is a direct target of NMD. Accordingly, in the presence of frameshift mutations, SMG1 inhibition induces the accumulation of truncated p53 proteins [62]. ATM, another protein involved in DNA damage response, is tightly regulated by NMD and its frameshift mutations result in loss of protein expression. Ataxia telangiectasia (AT) patients bearing this kind of mutations are hypersensitive to double-strand DNA breaks, bear neurological symptoms and are more likely to develop cancer. Interestingly, a similar phenotype is found also in patients characterized by mutations in other proteins involved in DNA damage sensing, such as MRE11, and which are targets of NMD-mediated degradation [63]. Hence, in the case of mutated p53, ATM and MRE11, NMD contributes to the worsening of the phenotype by reducing the levels of these already hypofunctional proteins and further impairing the DNA damage response pathway. Importantly, the expression levels of several genes involved in DNA damage response are regulated by NMD also in the absence of truncating mutations, but through mRNA splicing. The latter mechanism is linked to NMD since the splicing machinery is also responsible for the stability of mRNAs, it regulates basically each round of translation of an mRNA [64], and eventually controls the levels of proteins. EJC proteins are recruited at exon–exon junctions by the splicing machinery and are detached upon translation in the ribosome. If a PTC is present, the EJC remains on the mRNA and triggers NMD. Hence, splicing and NMD machineries are strictly interconnected, and work in concert during protein synthesis. Components of the splicing machinery are often mutated in cancer cells and are responsible for splicing aberrations and expression of 3′ cryptic transcripts. This is the case of splicing factor 3B subunit 1 (SF3B1; [65]) that is frequently mutated in chronic lymphocytic leukemia (CLL) resulting in aberrant response to DNA damage [66]. Notably, it has been shown that mutations in the splicesome genes SF3B1 and U2AF1 render cancer cells more sensitive to NMD inhibition in a synthetic lethality fashion [67]. Mutations in the splicesome and or NMD machinery may hence represent a predictive marker of response to NMD-directed compounds.

### Targeting the NMD pathway

In the presence of a dominant nonsense mutation, NMD reduces the expression of the mutated allele having so an important role in reducing the disease phenotypic manifestations [50]. In other cases, nonsense mRNA can result in the production of a truncated protein that is partially functional. Hence the destruction of this transcript by NMD results in a more severe phenotype [68]. The latter observation has provided the rationale for a promising therapeutic approach for genetic diseases driven by nonsense mutations [69] which consists of the use of small readthrough molecules that force ribosomes to ignore PTC and synthesize a full-length protein [70]. Approaches to prevent the degradation of nonsense transcripts by NMD coupled with readthrough agents have been tested in pathologies like cystic fibrosis (CF) and Duchenne muscular dystrophy (DMD). Aminoglycoside antibiotics were among the first to be used (Table 1). Thanks to their structure, they are able to bind ribosomes, reduce the fidelity of translation and, at the same time, prevent recognition of nonsense mRNA by NMD [78]. In vitro experiments in a CF bronchial epithelial cell line show that this double aminoglycoside effect allows an increase of about 20% of protein expression [79]. Despite this, clinical trials in patients with CF or DMD showed no therapeutic benefit, although aminoglycoside treatment led to increased functional protein production [80, 81]. Moreover, prolonged treatments with the high dose of aminoglycoside required to achieve clinical utility have been seen to cause severe adverse effects [82, 83]. In an attempt to identify new molecules with high efficiency and reduced toxicity, PTC Therapeutics Inc. performed a high throughput screen of about 800,000 molecules that allowed the identification of a small molecule named Ataluren or PTC124 [84]. Despite the acceptable safety profile obtained in preclinical studies, phase II and III trials for CF and DMD displayed conflicting results [85, 86] allowing Ataluren to be approved by FDA only for DMD treatment. These trials show also that individuals with the same genetic mutation have a different response to therapy, implying so the involvement of mutation-independent factors.

**Table 1:** Structure, mechanism of action and side effects of NMD pathway inhibitors

Molecule	Structure	Mechanism of action	Side effects
Aminoglycosides [71]		PTC readthrough	Toxic at clinical active concentrations
Caffeine [72]		SMG1 inhibition	Toxicity, highly aspecific by targeting several kinases
NMDI-1 [73, 74]		Stabilization of the phosphorylated form of UPF1	No side effects in mice
5-azacytidine [75]		Non identified, but c-MYC-mediated	Already approved for the treatment of diverse malignancies
Amlexanox [76]		Inhibits NMD at a late stage	Clinically approved for aphthous ulcers
NMDI14 [77]		Disrupts the SMG7–UPF1 complex	Negligible toxicity in cell-based assays

The regulation of the NMD pathway is extremely variable among the population and the level of degradation of nonsense mRNA, which are the target of readthrough molecules, is patient-dependent [87, 88]. Due to the complexity of NMD regulation, it is difficult to identify the specific determinants of the observed variability. NMD efficiency was shown to be cell- [89] and tissue-specific [90], and the same mutation was found to result in extremely variable intra- and inter-familial phenotypes [88]. Expression levels of the core components of the NMD machinery, which are themselves targets of the NMD! [91], could contribute to the variable NMD activity found [92]. Their transcription was shown to depend on a plethora of mechanisms including expression quantitative trait loci (eQTL), miRNAs, mRNA half-life and negative feedback loop (reviewed in [93]). Mutations can be found in genes coding for NMD mediators especially in cancer cells, [56, 94] and stress conditions further influence the levels and the activity of NMD [32]. Several reporters have been developed to estimate NMD activity in preclinical settings [92] and recent advances in whole cDNA sequencing could allow to quantify the whole activity of NMD and identify thousands of mRNAs targeted by NMD [24], exploitable as biomarkers to design a personalized approach and treat patients with NMD inhibitors for both genetic diseases and cancer.

Due to the inter-patient different sensitivity to treatment, the association between readthrough agents and NMD inhibitors is focus of several studies that demonstrate a synergistic effect in the rescue of nonsense mutations [95]. Despite the essential role of NMD in the cell, it has been shown that the depletion of UPF1 to up to 80 % allows NMD activity suppression without compromising the survival of cells, suggesting that the use of NMD inhibitors could display a therapeutic window [77].

Several compounds have been identified as NMD inhibitors, limiting the UPF1 phosphorylation and dephosphorylation cycle [96]. An example is constituted by caffeine that, through inhibition of SMG1, reduces the phosphorylation of UPF1 and so NMD activity. In vitro experiments on a bronchial cell line derived from a CF patient with a nonsense mutation show that caffeine reduces the NMD-dependent nonsense mRNAs degradation and allows an enhanced efficiency of readthrough [97]. Despite this evidence, caffeine, even if it does not affect cell viability and morphology, is not specific for SMG1 inhibition and inhibits also phosphoinositide-3-kinase related protein kinases (PIKKs) and some phosphoinositide 3-kinases (PI3Ks) [72, 98]. However, a region of about 1000 amino acids found in SMG1, which is not in common with other kinases, could provide a possible target for new target-specific NMD inhibitors [99].

Through a high throughput drug screen, a small molecule, referred to as NMDI-1, was identified and it was shown to be able to stabilize the hyperphosphorylated state of UPF1, so determining NMD inhibition. NMDI-1 was shown to be rather specific and induce low toxicity in mammalian cells, also when used at concentration higher than necessary [73]. In order to optimize the activity of this inhibitor, NMDI-1 derivatives (NMDI-9, NMDI-14, NMDI-25) have been developed and in vitro experiments show that their combination with readthrough agents allows to restore a functional full-length p53 protein [77]. Another possible NMD inhibitor is the 5-azacytidine (5AzaC), a cytidine analogue that, once incorporated into DNA, inhibits methyltransferases allowing so expression of genes, like tumor suppressor ones, previously suppressed by methylation. For this activity 5AzaC, is already approved by FDA for cancers treatment and so could be potentially repurposed as a therapeutic NMD inhibitor [100]. In particular, 5AzaC NMD inhibition is different from the classical already analyzed mechanisms and acts in a c-MYC dependent manner. In fact, 5AzaC anti-NMD activity is fully eliminated in a c-MYC knockdown context [75]. Although the precise mechanisms underlying the latter observation are largely unknown, c-MYC is linked to NMD via several mechanisms. In fact, it is a well-known inducer of protein synthesis, hence triggering UPR and phosphorylation of eIF2α, which can also be induced by replication stress and/or DNA damage again, all favored by c-MYC. Moreover, c-MYC induces ROS [101] another regulator of NMD. Notably, c-MYC is an oncogene often overexpressed in tumors that also exhibit a repressed NMD pathway.

Instead, a repurposing drug approach through virtual screening of FDA-approved drugs allowed the identification of a new promising NMD inhibitor, amlexanox. It is an anti-inflammatory and anti-allergic compound approved for the treatment of mouth ulcers and asthma, and moreover tested for a Phase II clinical trial for diabetes mellitus type II (www.clinicaltrials.gov). Amlexanox stabilizes nonsense mRNAs in a late stage of NMD pathway and so acts with a different mechanism from the other analyzed inhibitors [76, 102]. Moreover, in vitro experiments show that amlexanox treatment allows a more efficient rescue of mutated proteins compared to amynoglycosides and results can be appreciated already after 24 h from the treatment instead of the 48 h requested by aminoglycosides treatment [102]. The relative safety, together with its efficiency, makes Amlexanox a promising therapeutic molecule for NMD inhibition.

### Novel strategies to modulate NMD in cancer

Amlexanox has been proposed, at least in preclinical settings, to treat cancer cells suggesting that NMD inhibitors could be repurposed for colorectal, and especially MSI, cancer treatment to restore the expression of several NMD targets. Accordingly, it was shown that amlexanox administration decreases cell proliferation of MSI, but not microsatellite stable (MSS), cells [3] both in vitro and in vivo. Notably, only immunocompromised animals were employed supporting the idea that this approach could be even more effective in syngeneic immunocompetent models. In the presence of a competent immune system, the inhibition of NMD could allow the exposure of several mutated antigens inducing an anti-tumor response in the host. It is possible to speculate that there could be a correlation between TMB and/or mismatch repair-deficiency, and efficacy of NMD inhibition in cancer, as already observed for anti–PD-1 or anti–PDL1 therapy [103]. On the same line, pharmacologic perturbation of splicing could potentially be exploited in cancer treatment, as recently shown in mice models, to produce neoepitopes and trigger an anti-tumor immune response [104].

On the other hand, as already mentioned, NMD appears to be endowed with tumor suppressor activity in some tumors. In these settings, an activation of NMD would be preferable. Although a pharmacological approach to restore NMD activity is difficult to design, a possible strategy could be represented by the modulation of the diet. Caloric restriction has already been shown to enhance the efficacy of chemotherapy [105], hormone therapy [106] and immune checkpoint therapy [107], but it could also be effective in triggering the expression levels of NMD mediators through the stimulation of AS [108]. Hence, a number of drugs and interventions already available for the treatment of genetic diseases could be repurposed in cancer treatment to inhibit or, contrarily, activate NMD.

## Conclusions

NMD was initially identified as a mechanism to merely prevent the expression of mutated truncated proteins which have the potential to be toxic by displaying dominant negative activities. Later works have clarified that NMD is endowed with a more wide effect on basically every cellular aspect through its capability to control the stability of mutated and non mutated mRNAs. NMD function is also connected to the splicing machinery activity further increasing its capability to control cell physiology and adaptation to stress. NMD has been shown to play a crucial role in tumors although the intimate mechanisms and effects of NMD in cancer cells are still poorly characterized. Many works have proved a pro-tumor effect of NMD, but, at the same time, a large amount of evidence shows that NMD can display a tumor suppressor activity. Undoubtedly, NMD represents an opportunity for new cancer therapy strategies, pending the comprehension of the intricate setting-dependent features. The identification of predictive markers of response to NMD inhibition is hence crucial. An approach could be represented by quantification of NMD activity in cancer cells, which can be higher or lower compared to normal tissues in a patient-dependent fashion [109].

Patients bearing tumors characterized by high levels of NMD activity could theoretically benefit from molecules inhibiting one or more of its mediators. Several compounds are currently been tested for genetic diseases with the final aim of restoring, via NMD inhibition, the expression of mutated proteins that, although truncated, could preserve at least in part their activity and eventually reverting the aberrant phenotype. In cancer settings, NMD inhibition could promote the accumulation of cancer cell-specific neoantigens, potentially immunogenic (Fig. 1). Notably, these antigens could stem not only from frameshift mutations, but also from aberrant splicing which is particularly frequent in cancer cells [110, 111] and can produce novel, immunogenic peptides [112]. The inhibition of NMD could hence render tumors more immunogenic. This effect could be exploited by combination with immune checkpoint inhibitors and ultimately trigger or potentiate their activity [104]. In fact, despite the success of immune checkpoint inhibitors in several cancer types, their efficacy is often partial and there is the urgent need to find combinations to increase their efficacy in unresponsive patients and in cancer types not sensitive to this kind of therapy.

In conclusion, NMD may offer a novel opportunity in cancer treatment though it seems to display different and even opposite effects depending on the context. Still, the characterization of this pathway could support the designing of new strategies to treat tumors, especially in combination with immune-based therapies making mandatory the detailing of the intricate mechanisms controlling NMD and the identification of predictive markers which foresee the benefit of NMD inhibition.

## Data Availability

Not applicable.

## Abbreviations

5AzaC: 5-azacytidine
AS: Alternative splicing
AT: Ataxia telangiectasia
CF: Cystic fibrosis
CLL: Chronic lymphocytic leukemia
CRC: Colorectal cancer
DMD: Duchenne muscular dystrophy
eIF2α: eukaryotic translation initiation factor 2α
EJC: Exon junction complex
ESC: Embryonic stem cells
IMT: Inflammatory myofibroblastic tumor
MMR: Mismatch repair
MSI: Microsatellite instability
MSS: Microsatellite stable
NMD: Nonsense-mediated mRNA decay
PABC1: Cytoplasmic polyadenylate-binding protein
PI3K: Phosphoinositide 3-kinases
PIKK: Phosphoinositide-3-kinase related protein kinases
PTC: Premature termination codon
ROS: Reactive oxygen species
SF3B1: Splicing factor 3B subunit 1
SURF: SMG1-Upf1-eRF1-eRF3
TCR: T-cell receptor
TME: Tumor microenvironment
UPR: Unfolded protein response
